# Prognostic Value of Ezrin in Solid Tumors: A Meta-Analysis of the Literature

**DOI:** 10.1371/journal.pone.0068527

**Published:** 2013-07-19

**Authors:** Kun Han, WeiXiang Qi, ZhiHua Gan, Zan Shen, Yang Yao, DaLiu Min

**Affiliations:** 1 Department of Oncology, Shanghai Sixth People's Hospital East Campus, Shanghai Jiao Tong University, Shanghai, China; 2 Department of Oncology, The Sixth People's Hospital, Shanghai Jiao Tong University, Shanghai, China; INRS, Canada

## Abstract

**Purpose:**

Ezrin is a cytoskeletal protein involved in tumor growth and invasion. However its prognostic value for survival in patients with solid tumor remains controversial.

**Methods:**

Several databases were searched, including Pubmed, Embase and Cochrane databases. The endpoints were overall survival (OS), progression-free survival (PFS). The pooled hazard ratio (HR) or odds ratio (OR), and 95% confidence intervals (CI) were calculated employing fixed- or random-effects models depending on the heterogeneity of the included trials.

**Results:**

Twenty-seven eligible trials involving 4693 patients were ultimately identified. A summary hazard ratio (HR) of all studies and sub-group hazard ratios were calculated. The combined HR suggested that a positive Ezrin expression had an impact on overall survival (OS) [1.95, 95% confidence interval (CI) 1.60–2.39; P<0.001] in all eligible studies and progress free survival (PFS): (2.30 95% CI 1.0–3.61; P = 0.001). Similar results were also observed in subgroup analysis, according to tumor types, regions, patients' number and publication year.

**Conclusions:**

Our findings suggested that Ezrin protein expression might be a factor for a poor prognosis in patients with solid tumor. So large well-designed prospective studies are now needed to confirm the clinical utility of Ezrin as an independent prognostic marker.

## Introduction

Ezrin, a member of the ezrin/radixin/moesin (ERM) family, is an important molecule linking the cytoskeleton to the membrane [1]. Ezrin is essential for many fundamental cellular processes, including determination of the cell shape, polarity, surface structure, cell adhesion, motility, cytokinesis, phagocytosis, and integration of membrane transport through signaling pathways [1], [2], [3], all of which are expected to promote tumor progression. Indeed, recent studies have revealed that ezrin may have an important role in tumorigenesis, development, invasion, and metastasis, probably through regulation of adhesion molecules, participation in cell signal transduction, and signaling to other cell membrane channels in the tumor [4], [5]. For long a large number of studies have been focused on identifying the prognostic value of Ezrin in solid tumors and most studies suggest that Ezrin is beneficial for tumor growth and, therefore, associated with poor prognosis including carcinomas of the breast [6], soft tissue sarcoma [7], ovary cancer [8], Gastrointestinal stromal tumors [9],colorectal cancer [10] and non-small cell lung cancer [11]. In this study, we sought to conduct a meta-analysis to estimate the prognostic importance of Ezrin level for overall survival (OS) and disease-free survival (DFS) among patients with solid tumors, aiming to gain insights into whether Ezrin could provide useful guidance in the biological understanding and treatment of solid tumors.

## Materials and Methods

### Literature search

We conducted a comprehensive search in the Pubmed and Embase to include in the present meta-analysis. We combined search terms for Ezrin expression and solid tumors: (“solid tumor” or “solid cancer”) or “Ezrin” or “prognosis”. And the last search was updated on 31 Dec 2012. We also reviewed the Cochrane Library for relevant articles. The references cited in those included studies were also reviewed to complete the search.

Study was conducted according to the Preferred Reporting Items for Systematic Reviews and Meta-Analyses (PRISMA) statement [12].

### Inclusion and exclusion criteria

Inclusion criteria for this study were as follows: (1) proven diagnosis of solid tumor, (2) Ezrin evaluation using immunohistochemical method, (3) association of Ezrin with overall survival (OS), and/or disease-free survival (DFS). Reviews, letters to the editors, and articles published in a book were excluded. We avoided duplication of data by examining the names of all authors and medical centers involved for each article. Authors that published multiple reports on the same sample were included once. We did not weight each study by a quality score because no such score had received general agreement for meta-analysis of observational studies [13].

### Data extraction

Two independent reviewers (HK and QWX) read titles and abstracts of all candidate articles. Articles that could not be categorized based on title and abstract alone were retrieved for full-text review. Articles were independently read and checked for inclusion criteria of articles in this study. Any disagreement in quality assessment and data collection was discussed and solved together. The following data were collected: (1) article data including publication date, first author's name and country; (2) demographic data regarding inclusion criteria, age, regions, number of patients and number of Ezrin positive; (3) tumor data of Underlying malignancies; (4) survival data including OS, DFS and follow-up period; (5) method of Ezrin measurement, cut-off used for assessing Ezrin positivity. Any differences in the data extraction were resolved together by two authors.

### Statistical analysis

Hazard ratios (HRs) and its 95% confidence intervals (CIs) were used to estimate the association between Ezrin and patients' prognosis. For those HRs that were not given directly in the published articles, the published data including the number of patients at risk in each groups, the total number of events and figures from original articles were used to estimate the HR according to the methods described by Parmar et al [14]. If the only exploitable survival data were in the form of figures, we read Kaplan-Meier curves by Engauge Digitizer version 4.1 (free software down-loaded from http://sourceforge.net) and extracted survival rate from them to reconstruct the HR and its standard error (SE). All the data analyses were performed with Stata version 11.0 (Stata Corporation, College Station, TX, USA) and we used Q-tests and P-values to estimate the heterogeneity. If P-value was greater than 0.05 which indicated a lack of heterogeneity among studies, a fixed-effects model was used to calculate the HR and its 95%CI according to the method of Mantel and Haenszel [15]. Otherwise, a random-effects model (the DerSimonian-Laird method) was used. By convention, an observed HR>1 implied a worse prognosis in the Ezrin positive group. The impact of Ezrin on survival was considered to be statistically significant if the 95%CI for the HR did not overlap 1.

## Results

### Study selection and characteristics

**Figure 1:**
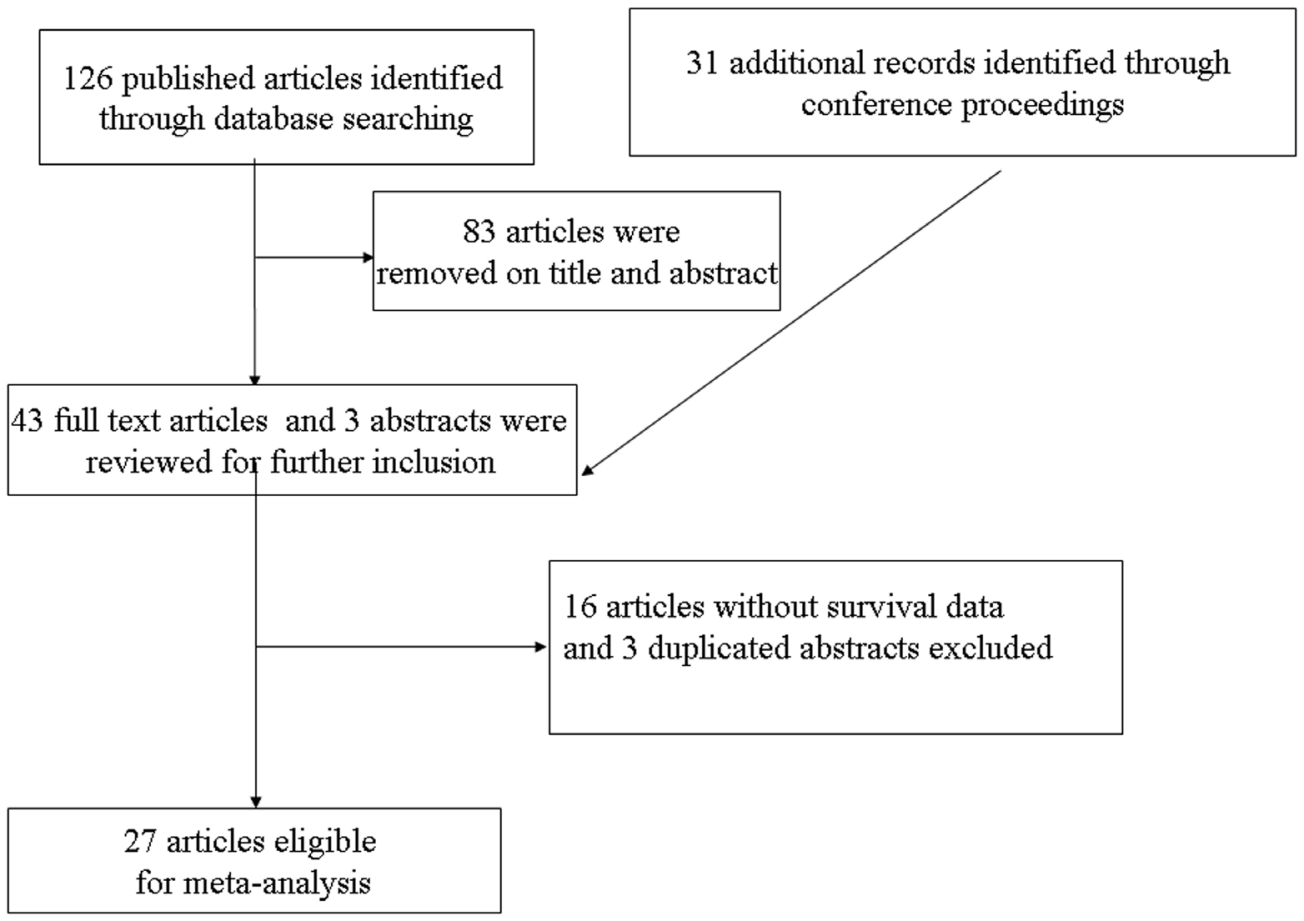
Methodological Flow Chart of the Systematic.

A total of 126 potentially relevant studies were retrieved electronically, 99 of which were excluded for the reasons shown in figure 1. Full-text copies of the remaining 43 citations were obtained and were evaluated in more detail. Finally, a total of 27 trials with 4693 patients were available for the meta-analysis.

The main features of the eligible studies for Ezrin were summarized in Table 1. The total number of patients included for meta-analysis was 4693, ranging from 40 to 487 per study. In total, 22 studies had data on OS [6], [7], [8], [10], [11], [16], [17], [18], [19], [20], [21], [22], [23], [24], [25], [26], [27], [28], [29], [30], [31], [32], and 7 study have data on DFS [6], [9], [23], [33], [34], [35], [36]. 13 reports originated from Asia, 14 from Non Asia. Number of positive patients ranged from 12 to 240 in the included 27 studies.

**Table 1:** Main Characteristics of the Eligible Studies.

Author	Year	Region	No of patients	Underlying malignancies	Technology	Positive (%)	Survival analysis	HR estimation	HR (95%)	Cut-off For Ezrin +
Makitie	2001	Finland	130	Uveal Malignant Melanoma	IHC	83	OS	HR	2.52 (1.4–4.51)	at least positive
Moilanen	2003	Finland	440	Ovarian carcinoma	IHC	318	OS	K-M	1.34 (1.06–1.62)	≥10%
Weng	2005	Sweden	50	soft tissue sarcomas	IHC	25	OS	K-M	1.48 (0.67–3.28)	>1%
Yeh	2005	Taiwan	84	pancreatic cancer	IHC	49	OS	K-M	1.36 (0.98–1.89)	at least moderate
Kobel	2006	Germany	105	ovarian carcinoma	IHC	51	OS	K-M	2.16 (1.31–3.55)	at least moderate
Kobel	2006	Germany	164	endometrioid carcinomas	IHC	83	OS	K-M	1.46 (0.52–4.14)	at least moderate
Madan	2006	USA	40	HNSCC	IHC	19	OS	HR	1.82 (1.0–3.2)	≥10%
Mhawech-Fauceglia	2007	Switzerland	108	HNSCC	IHC	93	DFS	HR	0.266 (0.63–1.111)	at least moderate
Elzagheid	2008	Finland	74	Colorectal cancer	IHC	61	OS	K-M	1.88 (0.81–4.36)	at least moderate
Gao	2009	China	193	esophageal carcinoma	IHC	90	OS	HR	1.46 (0.99–2.15)	≥50%
Palou	2009	Spain	92	bladder tumours	IHC	12	OS	K-M	7.21 (1.43–36.36)	>20%
					IHC		DFS	K-M	4.01 (1.10–14.62)	
Wei	2009	Taiwan	347	GIST	IHC	229	DFS	HR	2.363 (1.254–4.454)	≥50%
Huang	2010	Taiwan	78	myxofibrosarcomas	IHC	38	OS	K-M	3.13 (1.34–7.28)	at least moderate
Kang	2010	Korea	100	hepatocellular carcinoma	IHC	28	DFS	K-M	2.25 (1.41–3.57)	>10%
Aishima	2011	Japan	41	intrahepatic cholangiocarcinoma	IHC	20	OS	K-M	1.81 (0.92–3.55)	>11%
Carneiro	2011	Sweden	227	soft tissue sarcomas	IHC	109	DFS	HR	1.8 (1.1–2.8)	at least moderate
Korkeila	2011	Finland	176	Rectal cancer	IHC	15	DFS	K-M	3.26 (1.09–9.72)	at least moderate
Lam	2011	HongKong	150	Gastric cancer	IHC	117	OS	HR	2.016 (1.099–2.933)	at least moderate
Li	2011	China	436	Gastric carcinoma	IHC	236	OS	K-M	2.07 (1.35–3.19)	at least moderate
Patara	2011	Brazil	250	Colorectal cancer	IHC	21	OS	K-M	1.62 (0.75–3.49)	at least moderate
Wang	2011	China	200	nasopharyngeal carcinoma	IHC	134	OS	K-M	1.96 1.091–2.828)	at least moderate
Wang	2011	China	75	Salivary gland adenoid cystic carcinoma (SACC)	IHC	23	OS	HR	2.23 (1.02–4.9)	at least intense
Xie	2011	China	307	Esophageal	IHC	240	OS	K-M	1.47 (1.08–2.01)	at least moderate
Jorgren	2012	Sweden	104	Rectal cancer	IHC	86	OS	K-M	1.89 (1.16–3.1)	at least moderate
Lee	2012	Korea	112	NSCLCs	IHC	33	OS	HR	1.853 (1.053–3.623)	at least positive
Schlecht	2012	USA	123	HNSCC	IHC	34	OS	HR	3.11 (1.35–7.15)	≥10%
Ma	2013	China	487	Breast cancer	IHC	74	OS	HR	3.711 (3.112–4.371)	≥75%
							DFS	HR	3.805 (3.002–4.386)	

HR hazard ratio, K-M Kaplan Meier, OS overall survival, DFS disease free survival, IHC immunohistochemical.

### Publication bias

NoevidenceofpublicationbiaswasdetectedfortheHR of OS and PFS in this studybyeitherBeggorEgger'stest(HRofOS:Begg'stestp = 0.085,Egger'stestp = 0.455;HR of PFS: Begg'stestp = 0.293,Egger'stestp = 0.764) (Fig. 4 and Fig. 5).

### Meta-analysis

**Figure 2:**
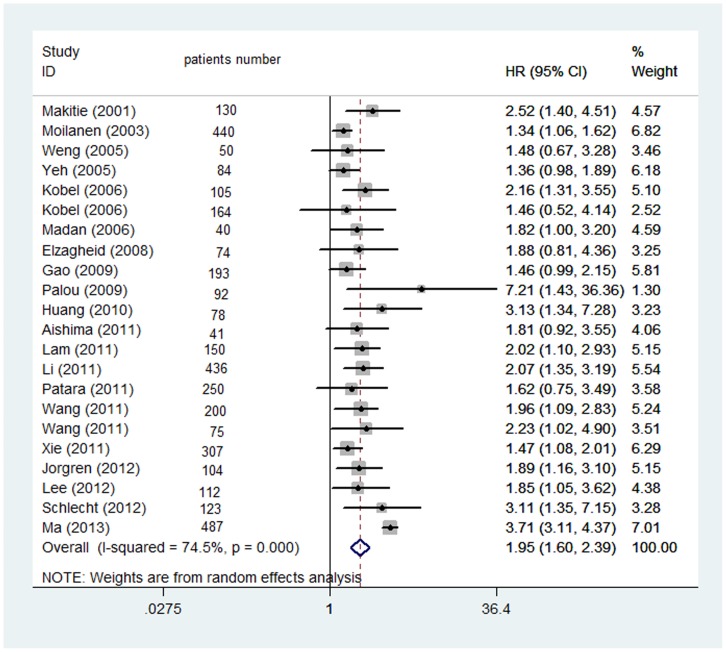
Ezrin expression and OS.

**Figure 3:**
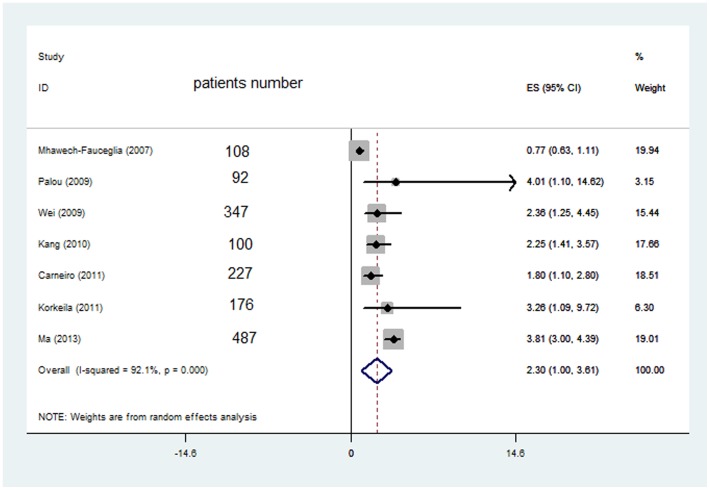
Ezrin expression and PFS.

The results of the meta-analysis were shown in Fig. 2 and Fig. 3. The combined HR for 22 studies evaluating Ezrin overexpression on OS was 1.95, (95% CI: 1.60–2.39), suggesting that Ezrin overexpression was an indicator of poor prognosis for solid tumor. Significant heterogeneity was observed among the studies. (Q = 55.4, I2 = 62.1%, P<0.001). When grouped according to geographic settings of individual studies, the combined HRs of Asian studies and non-Asian studies were 2.006 (95% CI: 1.483–2.529) and 1.498 (95%CI: 1.260–1.735) respectively. Subgroupanalysiscouldhelpusdiscoverpotentialinformation of what the clinicians were interested in. Therefore, we studied some factors that might be related with survival. The studies from the tumor types, regions, patients' number and publication year were considered as the subgroup analysis factors. Finally, all subgroup analyses favored Ezrin overexpression be associated with poor OS (Table 2). 7 studies evaluating Ezrin overexpression on PFS was 2.30, (95% CI: 1.00–3.61), indicate that Ezrin overexpression was an indicator of poor prognosis for solid tumor using random effect model(Q = 96.05, I2 = 92.1%, P<0.001).

**Figure 4:**
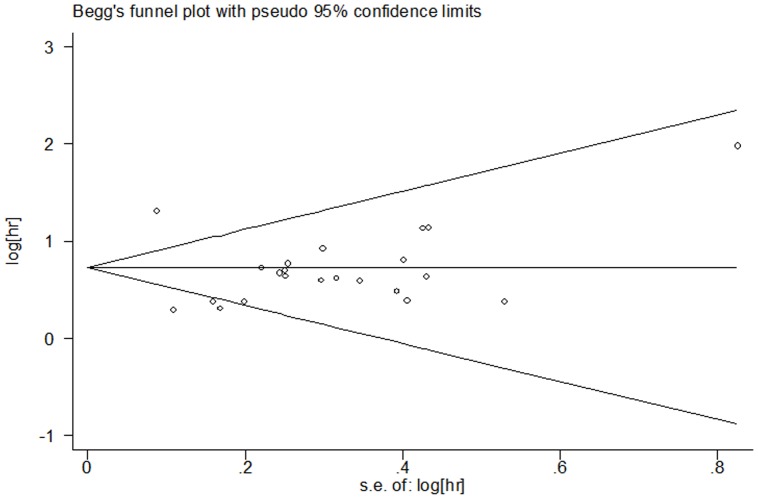
Begg's test result of OS.

**Figure 5:**
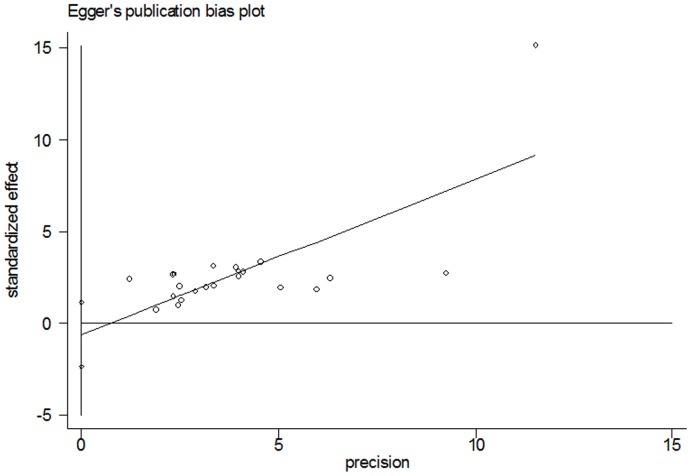
Egger's test result of OS.

**Table 2:** Stratified analysis of pooled hazard ratios of cancer patients with Ezrin expression.

Stratified analysis	No. of studies	No. of patients	Pooled HR (95%CI)	Heterogeneity	
				I^2^%	p-value
**Tumor type**					
**Head and neck cancer**	5	568	2.070(1.488–2.652)	0%	0.894
**Digestive cancer**	9	1639	1.565(1.325–1.806)	0%	0.871
**Other types**	8	1478	2.255(1.131–3.379)	87.3	<0.001
**Region**					
**Asian**	11	2163	2.006(1.483–2.529)	77.1	<0.001
**Non Asian**	11	1572	1.498(1.260–1.735)	0%	0.726
**No. of patients**					
**≥150**	9	2627	1.518(1.320–1.717)	33.5	0.150
**<150**	13	1108	1.694(1.386–2.002)	0%	0.874
**Publication year**					
**<2009**	10	1372	1.437(1.232–1.642)	0%	0.819
**≥2010**	12	2363	2.150(1.611–2.688)	67.9	<0.001

## Discussion

Ezrin is a member of the ERM (Ezrin, Radixin and Moesin) family, which was first described as linkers between membrane proteins and actin filaments. It has been implicated in the determination of cell shape, membrane organization, cell polarization, migration, division and they participate in various signaling pathways [5], [37], [38]. Alterations of ezrin expression can mediate many changes in the metastasis-associated cell surface signals and intra-cellular signaling cascade that confer the metastatic capability in tumor cells. Therefore, it is conceivable that ezrin overexpression and/or deregulation could contribute to the metastatic behaviors of tumors. Evidence from both animal models and prospective human studies show correlations between ezrin expression levels and tumor progression [37], [39], consistent with a crucial role for ezrin in tumor dissemination.

Meta-analysis is useful to integrate results from independent studies for a specified outcome. Pooled results from the combining relevant studies are statistical powerful, and make it possible to detecting effects that may be missed by individual studies.To date, no meta-analysis has been undertaken for any studies that evaluate Ezrin as a prognostic marker in solid tumor. In this meta-analysis, 27 eligible studies that compared the survival of solid tumor according to Ezrin expression level of the primary tumor met the enrollment criteria. The data were organized according to disease-free and overall survival; then combined results demonstrated that Ezrin overexpression was associated with a poor OS (HR, 1.95; 95%CI, 1.60–2.39; P<0.001.) and PFS (HR, 2.30; 95%CI, 1.00–3.61; P = 0.001.) in solid tumor using a random effect. Due to significant heterogeneity among included studies, we then perform a subgroup analysis according to tumor types, regions, patients' number and publication year. Allsubgroup analysesfavoredEzrin overexpression be associated with poorOS. In all our data helped to clarify the results of individual studies and to identify patients at high risk for whom specific- or adjuvant-therapy might be necessary since Ezrin overexpression is a prognostic factor for solid tumor.

There is significant heterogeneity among included studies in this systematic review, although we used random-effects models during pooling data of subgroup. The heterogeneity in these studies could be explained by different characteristics of included patients, or differences in the techniques used to detect alterations in Ezrin expression, including antigen retrieval methods, choice of Ezrin antibody, dilutions of the antibodies, and revelation protocols. What's more, different sample types including tissue microarray (TMA) and the whole section might also contribute to the heterogeneity because it is possible that more false-negative cases are obtained in TMA than the whole section. Finally, the differences of methodology among included studies also were sources of heterogeneity and caused selection biases potentially [40].

Several important limitations need to be considered when interpreting our analysis. First of all, the number of included studies was relatively small with only about 4693 cases. Patients had received different treatments; preoperative TNM category and histologic types were various. Whereas, we were unable to assess these potential confounders present in individual studies. Second, although we tried to identify all relevant data, potential publication bias was unavoidable and some data could still be missing. Third, although immunohistochemistry was the most commonly applied method for detecting Ezrin in situ, RT-PCR method had also been used for the evaluation of the levels of Ezrin gene or mRNA expression in tumor tissue. Studies measuring Ezrin gene or mRNA level by RT-PCR was not yet included in this meta-analysis. Moreover the cutoff value was defined differently (1%, 10%, 20%, 50%, 75%) in these studies, leading to between-study heterogeneity. Thus we had adopted random effect model and subgroup sensitivity analyses to adjust for the shortcomings.

Finally,this study was constrained to studies published in English language .Although we detected no evidence of publication bias using the graphical method, it was difficult to completely rule out this possibility.

In summary, this present study shows a significant correlation between Ezrin expression and OS as well as DFS rate in solid tumor patients. Ezrin may have prognostic significance for patients with solid tumor based on currently obtained data.However,one should be cautious when interrupting these results due to the limitations of our studies.Further high-quality studies are still needed to confirm these results.
